# Childhood emotional abuse and problematic social networking sites use in a sample of Italian adolescents: The mediating role of deficiencies in self‐other differentiation and uncertain reflective functioning

**DOI:** 10.1002/jclp.23138

**Published:** 2021-04-10

**Authors:** Alessandro Musetti, Vladan Starcevic, Valentina Boursier, Paola Corsano, Joël Billieux, Adriano Schimmenti

**Affiliations:** ^1^ Department of Humanities, Social Sciences and Cultural Industries University of Parma Parma Italy; ^2^ Faculty of Medicine and Health, Sydney Medical School, Nepean Clinical School, Discipline of Psychiatry University of Sydney Sydney New South Wales Australia; ^3^ Department of Humanities University of Naples “Federico II” Napoli Italy; ^4^ Institute of Psychology University of Lausanne, Geopolis Lausanne Switzerland; ^5^ Centre for Excessive Gambling Lausanne University Hospitals (CHUV) Lausanne Switzerland; ^6^ Faculty of Human and Social Sciences UKE—Kore University of Enna, Cittadella Universitaria Enna Italy

**Keywords:** adolescence, childhood emotional abuse, problematic social networking sites use, reflective functioning, self‐other differentiation

## Abstract

**Objective:**

Childhood emotional abuse (CEA) is associated with various negative mental health outcomes. This study aimed to investigate the association between CEA and problematic social networking site (SNS) use in a sample of Italian adolescents.

**Design:**

Using structural equation modeling, the study examined whether the relationship between CEA and problematic SNS use was sequentially mediated by self‐other differentiation and uncertain reflective functioning in 1308 Italian adolescents (628 males, age range 13–19 years).

**Results:**

A history of CEA was positively associated with problematic SNS use. Furthermore, deficiencies in self‐other differentiation and uncertain reflective functioning partially mediated the relationship between CEA and problematic SNS use.

**Conclusions:**

The present study provides additional insight into the psychological dynamics underpinning problematic SNS use among adolescents. The clinical implications of these findings are discussed.

## 1 INTRODUCTION

Social networking sites (SNS) have progressively become one of the most widely used means of online communication (Hussain & Starcevic, 2020). These types of social media (e.g., Facebook, Twitter, Instagram, and Snapchat) have been defined as “a networked communication platform in which participants (1) have uniquely identifiable profiles that consist of user‐supplied content, content provided by other users, and/or system‐provided data; (2) can publicly articulate connections that can be viewed and traversed by others; and (3) can consume, produce, and/or interact with streams of user‐generated content provided by their connections on the site” (Ellison & Boyd, 2013, p. 158). Nowadays, SNS use constitutes an important part of everyday adolescent life (Kuss & Griffiths, 2017) and plays a key role in providing social support for positive developmental trajectories (Lee & Horsley, 2017; Throuvala et al., 2019) and affirmation of identity (e.g., by increasing adolescents' self‐confidence in online self‐presentation among peers; Boursier & Manna, 2018). Accordingly, there are clear advantages of using social media for relationships (e.g., by maintaining existing relationships, optimizing communication, and providing up‐to‐date information; Horzum, 2016) and to help satisfy the need for relatedness and belonging (Nadkarni & Hofmann, 2012). However, SNS use has also been associated with certain risks in young people (Livingstone, 2008). Numerous studies have documented that a minority of people display problematic SNS use, which is defined as an excessive pattern of SNS use associated with negative consequences (e.g., interpersonal conflicts, interferences with sleep or work) and addiction‐like symptoms, such as loss of control, salience, tolerance, and withdrawal (Brand et al., 2016). In support of the clinical relevance of the phenomenon, various psychiatric disorders and maladaptive outcomes have been found to co‐occur with problematic SNS use, particularly in adolescents (Hussain & Griffiths, 2018). They include depression (Shensa et al., 2017; Worsley et al., 2018), anxiety (Andreassen et al., 2016; Ruggieri et al., 2020), stress (Müller et al., 2016; Worsley et al., 2018), poor family functioning (Wartberg et al., 2020), difficulties with emotion regulation (Marino et al., 2020), low self‐esteem (Saiphoo et al., 2020), and insecurity about one's own body image (Gioia et al., 2020).

Research has identified a variety of motives for frequent use of SNS by adolescents and young adults (e.g., socializing, self‐presentation, and acquiring information; Kitamura et al., 2019; Masur et al., 2014). Importantly, high SNS involvement is not necessarily associated with addictive patterns of SNS usage (Bashir et al., 2017). Although the distinction between various motives associated with frequent or problematic SNS use is still debated (Moreau et al., 2015), a recent study by Marino, Mazzieri, et al. (2018) showed that motives with negative valence (coping and conformity) were more closely associated with problematic use of Facebook than motives with positive valence (positive affect enhancement and improving relationships with friends). This is consistent with the compensatory model of problematic Internet use (PIU) (Kardefelt‐Winther, 2014a), which posits that motivations for problematic online activities reflect unmet needs or psychosocial problems. From this perspective, individuals may engage in online activities as a coping strategy to alleviate negative feelings and stressful life circumstances, despite these activities potentially leading to problematic use and negative consequences, such as psychological distress and poor psychosocial well‐being (Billieux et al., 2015; Boursier et al., 2020; Di Blasi et al., 2019; Kardefelt‐Winther, 2014b; Kuss & Billieux, 2017; Wéry et al., 2018). In other words, the compensatory model of PIU posits a vicious cycle, which starts with the person using SNS to escape from unpleasant feelings or events and a sense of being unable to cope with problems offline. This, in turn, can fuel excessive and ultimately problematic SNS use, which exacerbates the negative affect and the sense of being unable to cope. Self‐regulation is still in development during adolescence (Berthelsen et al., 2017; Pokhrel et al., 2013), and the emotional state of adolescents is often characterized by instability and negative affect (Larson et al., 2002). Therefore, individuals in this developmental stage could be more prone to coping with negative affect by using potentially problematic strategies, such as excessive SNS use (Kircaburun et al., 2019a).

Adding to this picture, an increasing body of research demonstrates that the detrimental effects of childhood trauma, including higher levels of negative affect and emotional instability (Sudbrack et al., 2015), may play a role in PIU (e.g., Schimmenti et al., 2014, 2017). However, relatively little is known about the relationships between these adverse experiences and problematic SNS use (Worsley et al., 2018). Given that Internet addiction is an “umbrella term” (Musetti et al., 2016; Starcevic & Billieux, 2017) that includes distinct forms of problematic online activities, such as problematic SNS use (Baggio et al., 2018), paying specific attention to the antecedents of problematic SNS use may advance the knowledge in this important field (Allen et al., 2014).

### 1.1 Childhood emotional abuse (CEA) and problematic use of SNS

CEA is a form of emotional maltreatment in childhood that refers to a consistent pattern of interactions between a caregiver and a child that result in psychological harm to the child (e.g., humiliation, excessive criticism, threat, and ridicule), excluding sexual or physical abuse (Hart et al., 1987). Although studies show a high rate of emotional maltreatment of children (e.g., Didie et al., 2006), most research has focused on physical and sexual abuse (Hagborg et al., 2017). Therefore, further research is needed to disentangle the relationship between specific forms of traumatic childhood experiences (i.e., physical, sexual, and emotional abuse and physical and emotional neglect) and various addictive behaviors (Dalbudak et al., 2014).

CEA is often associated with child emotional neglect but differs from it (Baker & Festinger, 2011). CEA refers to parental failures of care by commission, where parents actively and voluntarily humiliate and degrade the child. In contrast, childhood emotional neglect refers to parental failures of care by omission, where parents fail to meet the child's need for support, attention, affection, and care (Bifulco et al., 2002; Cicchetti & Toth, 2005; Infurna et al., 2016).

Research suggests that CEA often leads to further traumatization (Bifulco & Schimmenti, 2019; Schimmenti, 2018) and subsequent psychopathology (Midolo et al., 2020). Interestingly, Dalbudak et al. (2014) found that CEA was the most important predictor of PIU severity of all traumatic experiences in childhood. Moreover, a study of 11,956 European adolescents showed that adolescents without emotional and psychological support exhibited the highest risk of PIU (Durkee et al., 2012). A finding by Emirtekin and colleagues (2019) that CEA and CEN were positively associated with problematic smartphone use in adolescents is also important, considering that most people access SNS using smartphones (Gao et al., 2018). Moreover, in Kircaburun et al.'s (2020) study on cyberbullying perpetration among emerging adults, a positive association was found between childhood emotional maltreatment (i.e., CEA and CEN) and problematic social media use.

To the best of our knowledge, only one study to date has reported that adolescents with a history of emotional maltreatment may be more likely to develop problematic SNS use. Specifically, Kircaburun et al. (2019a) conducted a cross‐sectional study with a sample of 384 adolescents and found that childhood emotional maltreatment (i.e., CEA and CEN) was indirectly linked with problematic social media use via body image dissatisfaction among males, but not females. These findings suggest the need for developmentally informed research to better understand the relationship between CEA and problematic SNS use.

### 1.2 CEA, self‐other differentiation, and problematic use of SNS

Several studies (Hsieh et al., 2016; Worsley et al., 2018) suggest that problematic SNS use might be a maladaptive coping strategy for young people who have had traumatic childhood experiences. Accordingly, emotion dysregulation has been widely investigated as a mediator between childhood emotional maltreatment and addictive online behaviors (e.g., Evren et al., 2019; Kircaburun et al., 2019b; Lim et al., 2020). However, CEA not only has a negative impact on children's ability to regulate their emotions but could also undermine their developing sense of self (Bromberg, 2011) and their reflective functioning ability (Fonagy et al., 2002), that is, their capacity to understand and represent their own and others' mental states. Indeed, CEA has been associated with confusion about interpersonal roles, weak boundaries between parents and children (Kerig, 2005), and problems with self‐definition and self‐worth (Gladstone et al., 2004).

The acquisition of a stable sense of self and clear boundaries between oneself and others is one of the main developmental tasks that must be completed during adolescence (Blos, 1967; Majorano et al., 2015). Self‐other differentiation refers to one's capacity to experience a sense of self as a separate individual in their relationships with others (Olver et al., 1989). The formation of clear boundaries with others allows individuals to feel their uniqueness while at the same time enabling them to experience emotional intimacy with another person without fear of boundary dissolution (Kerig, 2005; Kerr, 1988). Individuals without a well‐differentiated sense of self tend to accept and interiorize the judgments, evaluations, interests, and orientations of others (Olver et al., 1989; Skowron & Friedlander, 1998). Previous research has already demonstrated that adolescents' problems with separation from parents and with individuation may be associated with PIU. Thus, Musetti, Corsano, et al. (2020) have found that adolescents' feelings of loneliness toward parents are linked to PIU via emotional detachment from parents, which is an unhealthy occurrence that takes place during the separation–individuation process. However, the link between self‐other differentiation and problematic SNS use has not been examined yet.

### 1.3 CEA, uncertain reflective functioning, and problematic use of SNS

CEA has a negative impact on the development of reflective functioning (Penner et al., 2019). Reflective functioning is the manifestation of the ability to mentalize (Fonagy et al., 2002), which refers to one's capacity for understanding the mental states (i.e., thoughts, feelings, wishes, and desires) underlying human behavior. Safe early relationships with parents or caregivers that are based on emotional attunement establish the primary regulatory parameters for self‐organization with a sense of a separate self (Adshead, 2008; Fonagy & Target, 1997). Reflective functioning reduces dependence on others for understanding and interpreting interpersonal situations by attributing meaning to the personal experiences and behaviors of other people (Fonagy, Target, Steele, et al., 1998). Thus, reflective functioning is a basic facet of human interaction and contributes to the development of stable interpersonal networks (Sundqvist, 2010). Conversely, uncertain reflective functioning has been associated with emotion dysregulation (e.g., Morosan et al., 2020); difficulties with impulse control, self‐monitoring, and self‐agency (Cosenza et al., 2019; Fonagy & Target, 1998); alexithymia (Badoud et al., 2015); and various forms of psychopathology (Luyten et al., 2020), including substance abuse and addictive disorders (Ciccarelli et al., 2020; Handeland et al., 2019; Möller et al. (2017). With regard to addictive behaviors, one cross‐sectional study of 466 adolescents showed that a history of traumatic childhood experiences and uncertain reflective functioning were associated with higher levels of problematic mobile phone use, with females spending more time using mobile phones for social networking (Musetti, Brazzi, et al., 2020). To date, however, there is a lack of available studies on the relationship between CEA, reflective functioning, and problematic SNS use.

### 1.4 CEA, self‐other differentiation, uncertain reflective functioning, and problematic use of SNS

CEA, inadequate self‐other differentiation, and uncertain reflective functioning are all potential antecedents of problematic SNS use. According to the attachment theory, problematic SNS use may be rooted in early developmental processes and related to specific insecure attachment styles (D'Arienzo et al., 2019). Attachment theory posits that parent‐child attachment relationships are based on an interplay between self‐other differentiation (i.e., need for exploration) and self‐other relatedness (i.e., need for closeness) (Ainsworth & Bell, 1970; Bowlby, 1988). A child who has a secure attachment with their parents can develop adequate reflective functioning capacity (Fonagy et al., 2002; Gergely et al., 2002). Emotionally abused children tend to avoid being in close physical proximity to others or display an ambivalent attitude toward others when distressed because they have learned that a “secure base” (i.e., a parent or caregiver as a source of safety and comfort) is not unconditionally available or that a parental or caregiver figure can be hostile (Howe, 2006). Prior research has shown that there is a link between problems related to self–other boundaries (i.e., identity diffusion) and uncertain reflective functioning (De Meulemeester et al., 2017; Fonagy et al., 2016). These variables are closely related because the ability to differentiate between self and others is an early developmental milestone that precedes self‐recognition (Colonnello et al., 2013). In fact, Fonagy and Target (1996) introduced the concept of “psychic equivalence mode” to describe the pre‐reflective mental mode in which individuals equate inner and outer reality. Individuals who are not able to discriminate between outer reality and their inner mental states tend to exhibit an uncertain reflective functioning, which is manifested by a rigid and deficient understanding of mental states (Handeland et al., 2019). Such individuals are likely to perceive stressful events as disorganizing because of an impairment in reflective functioning capacity (Fonagy et al., 2002). Based on these considerations, we suggest that deficiencies in self‐other differentiation and uncertain reflective functioning may exert a sequential mediating effect between CEA and problematic SNS use.

### 1.5 The present study

The mediating factors involved in the relationship between CEA and online addictive behaviors continue to be debated in the literature. According to psychodynamic attachment‐oriented research, online addictive behaviors may develop as an attempt to cope with a dysregulated sense of self and painful mental states (Schimmenti & Caretti, 2010, 2016; Schimmenti et al., 2017). Indeed, emotionally abusive environments leave adolescents alone to cope with their disorganized states of mind, and consequently, their problematic SNS use may be a maladaptive coping strategy (Hsieh et al., 2016) or a way to find an alternative social network (Worsley et al., 2018). Given that adolescents with CEA often exhibit deficiencies in self‐other differentiation and reflective functioning impairments, these factors constitute plausible mediators in the relationship between CEA and problematic SNS use.

**Figure 1:**
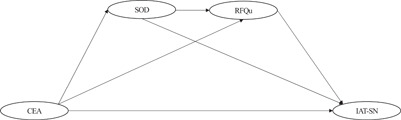
The hypothesized mediation model. CEA, childhood emotional abuse; IAT‐SN, Internet addiction test social network; RFu, reflective functioning‐uncertainty; SOD, self‐other differentiation

To the best of our knowledge, no published study has investigated the relationship between CEA and problematic SNS use while taking self‐other differentiation and uncertain reflective functioning into account. This led us to undertake the present study. We hypothesized that the severity of problematic SNS use would be positively associated with the severity of CEA and that this relationship would be partially and negatively mediated by adequate self‐other differentiation and, in sequence, partially and positively mediated by uncertain reflective functioning. The multiple mediation model configuring the postulated relationships between the variables is shown in Figure 1.

## 2 METHOD

### 2.1 Participants

The study included 1308 adolescents aged between 13 and 19 years (*M* = 16.27, *SD* = 1.48). Participants were recruited from nine high schools specializing in different areas of study, such as humanities, science, technology, accountancy, and art. The schools were located in three urban areas of the Emilia Romagna region in Italy. Females constituted 680 participants, and 1197 were of Italian origin. The immigrant adolescents came from Eastern Europe (*n* = 39), North Africa (*n* = 33), Asia (*n* = 13), and South America (*n* = 4), with 22 not reporting their country of origin. Regarding family status, 1,075 adolescents lived with married parents. With respect to parents’ education, the mothers of 683 participants and the fathers of 654 participants had completed upper secondary education.

### 2.2 Procedure

The head teachers of each school were contacted to request permission to collect data from the students. A presentation letter explaining the aims of the study and the procedures was sent to the families of the students. The students’ parents or legal guardians had to sign the informed consent to allow their participation, which consisted of their completion of the questionnaires in the students’ classrooms. Confidentiality and anonymity were rigorously ensured by assigning code numbers instead of names on all questionnaires. Data were collected between September 2019 and December 2019. Upon completion, all participants were thanked and debriefed by research assistants and were given an opportunity to ask questions. Students did not receive any compensation for participation. Data obtained from questionnaires that were not completed adequately and in their entirety were excluded from the analyses. The study was designed and carried out according to the Ethical Code of the Italian Association of Psychology (AIP), the European Code of Conduct for Research Integrity (ECCRI), and the 1964 Helsinki Declaration and its later amendments. The data presented in this study are part of a larger investigation on the relationship between childhood trauma and addictive behaviors among adolescents (Musetti, Brazzi, et al., 2020).

### 2.3 Measures

#### 2.3.1 Internet Addiction Test‐Social Networking Site (IAT‐SNS)

The Italian version of the IAT (Ferraro et al., 2007; original version by Young, 1998) was adapted for SNS use following previous studies (Romo et al., 2017; Rothen et al., 2018). The scale consists of 20 items (e.g., “How often do you find that you stay on social networking sites longer than you intended?”), which are rated on a five‐point Likert scale from 1 (*never*) to 5 (*always*). There are no reverse‐scored items. The total score is obtained by summing all items (score range = 20–100), where higher values indicate higher levels of problematic involvement in SNS. Participants whose total IAT‐SNS score was above 50 were classified as being at risk of problematic SNS use (e.g., Musetti et al., 2018; Schimmenti et al., 2012). Scores were also calculated on six subscales of the IAT‐SNS: (1) Salience (five items, e.g., “How often do you fear that life without SNS would be boring, empty, and joyless?”), which measures preoccupation with SNS use; (2) Excessive Use (5 items, e.g., “How often do you find that you stay on SNS longer than you intended?”), which reflects excessive or compulsive SNS use; (3) Neglect Work (three items, e.g., “How often does your school performance or productivity suffer because of SNS use?”), which measures impairment in school or work performance due to the amount of time spent on SNS; (4) Anticipation (two items, e.g., “How often do you find yourself anticipating when you will go on SNS again?”), which reflects a pervasive need for SNS use; (5) Lack of Control (three items, e.g., “How often do you find yourself saying ‘Just a few more minutes,’ when you are using SNS?”), which reflects difficulty managing and controlling SNS use; (6) Neglect Social Life (three items, e.g., “How often do you prefer the excitement of SNS to spending time with friends?”), which assesses a tendency to use relationships established via SNS to cope with stress and offline problems. Cronbach's *α* for the IAT‐SNS in this study was 0.86.

#### 2.3.2 Emotional Abuse (EA)

The EA subscale of the Italian version of the Childhood Trauma Questionnaire‐Short Form (CTQ‐SF; Sacchi et al., 2018; original version by Bernstein et al., 2003) was used to assess the participants’ overall perception of emotional abuse during childhood. The EA subscale comprises five items (e.g., “When I was growing up, I thought my parents wished I had never been born”) rated on a 5‐point Likert scale ranging from 1 (*never true*) to 5 *(very often true*). The total score on the EA subscale is calculated by summing all items (score range = 5–25), with higher scores indicating higher levels of EA. There are no reverse‐scored items. Following previous studies (e.g., Kim et al., 2013; Mørkved et al., 2018), EA scores were dichotomized into none‐to‐low levels of CEA (score range = 5–12) and moderate‐to‐severe levels of CEA (score range = 13–25) (Bernstein & Fink, 1998). In the present study, the EA subscale showed a good internal consistency (Cronbach's *α* = 0.73).

#### 2.3.3 Self‐Other Differentiation Scale (SODS)

The Italian version of the SODS (Ingoglia et al., 2018; original version by Olver et al., 1989) was administered to evaluate the degree to which adolescents experience a separate sense of self in their relationships with others. The SODS comprises 11 items (e.g., “If someone close to me finds fault with what I do, I find that my self‐evaluation is lowered”) rated in a true‐false (0, 1) format. There are no reverse‐scored items. Participants were requested to read each item and decide whether or not it described them at the present time. A total score ranges from 0 to 11, with higher scores indicating higher levels of self‐other differentiation. In the present study, Cronbach's *α* was 0.70.

#### 2.3.4 Reflective Functioning‐Uncertainty (RFQu)

The RFQu subscale of the Italian version of the Reflective Functioning Questionnaire (RFQ; Morandotti et al., 2018; original version by Fonagy et al., 2016) was administered to assess participants' uncertainty about mental states. The RFQu consists of six items (e.g., “Sometimes I do things without really knowing why”; Cronbach's *α* in this study = 0.64), which are rated on a 7‐point Likert scale ranging from 1 (*totally disagree*) to 7 (*totally agree*). One item is reverse‐scored (i.e., “I always know what I feel”). A high score on the RFQu indicates a lack of understanding of internal mental states, including thoughts, emotions, and needs.

### 2.4 Data analytic strategy

Data were analyzed with IBM SPSS (version 24) and Mplus software (version 8). Descriptive statistics were computed for all variables. Normality assumptions were tested by skewness and kurtosis coefficients. Independent *t *tests were used to examine gender differences in CEA, SOD, RFQu, and problematic SNS use scores. The effect sizes of these differences were evaluated with Cohen's *d*, where ≥0.80 indicates a large effect, 0.50–0.79 indicates a moderate effect, and 0.20–0.49 indicates a small effect (Cohen, 1988). Intercorrelations were evaluated using Pearson correlation coefficients. Due to the expected correlations between the independent variables, multicollinearity was screened for by examining the variance inflation factors (VIF). No significant multicollinearity issues were identified (i.e., VIF = 1.05 for CEA, VIF = 1.20 for SOD, and VIF = 1.21 for RFQu, respectively). Subsequently, structural equation modeling (SEM) was used to test the sequential mediating effects of self‐other differentiation and reflective functioning uncertainty in the relationship between CEA and problematic SNS use. Latent variables for CEA and reflective functioning uncertainty were computed using items as indicators, whereas the latent variable for problematic SNS use was calculated using the six subscales of the IAT‐SN. To reduce model complexity (i.e., the SODS comprises 11 items), three parcels were used as indicators of the latent factor of SOD (Bandalos & Finney, 2001). These three parcels consisted of randomly selected SODS items (Matsunaga, 2008). The goodness of fit of SEM was evaluated using the root‐mean‐square error of approximation (RMSEA), the comparative fit index (CFI), and the standardized root‐mean‐square residual (SRMR). Hu and Bentler's (1999) goodness‐of‐fit criteria were employed to indicate acceptable (CFI and Tucker–Lewis index [TLI] > 0.90, SRMR < 0.10, RMSEA < 0.08) and excellent fit (CFI and TLI > 0.95, SRMR < 0.08, RMSEA < 0.06).

## 3 RESULTS

Descriptive statistics for the full sample and the sample differentiated by gender are reported in Table 1. One hundred participants (7.64%) showed a moderate‐to‐severe level of CEA (EA scores above 12). With regard to SNS use, 301 participants (23.01%) reported less than 2 h of average daily use, 715 (54.66%) reported 2–4 h of average daily use, and 292 (22.32%) reported more than 4 h of average daily use. In addition, 187 participants (14.30%) scored above 50 on the IAT‐SN and were therefore identified as being at risk for problematic SNS use.

**Table 1:** Descriptive statistics and gender differences for all investigated variables

	Overall, *M* (±*SD*)	Skewness	Kurtosis	Males (*n* = 628), *M* (±*SD*)	Females (*n* = 680), *M* (±*SD*)	Ranges	*t*(1310)	*p*	95% CI	*d*
CEA	7.1 (±3.19)	2.21	5.67	6.97 (±2.91)	7.22 (±3.43)	5–25	−1.43	0.15	[−0.60, −0.09]	0.08
SOD	6.69 (±2.64)	−0.23	−0.71	7.39 (±2.51)	6.05 (±2.56)	0–11	9.51	<0.001	[1.07, 1.63]	0.53
RFu	22.67 (±7.31)	−0.04	−0.32	21.83 (±7.35)	23.46 (±7.20)	6–42	−4.04	<0.001	[−2.45, −2.45]	0.23
IAT‐SN	39.92 (±10.83)	0.68	0.56	38.50 (±10.96)	41.22 (±10.56)	20– 91	−4.56	<0.001	[−3.85, −1.52]	0.24

Abbreviations: CEA, childhood emotional abuse; CI, confidence interval; *d*, Cohen's *d*; IAT‐SN, Internet addiction test social network; RFu, reflective functioning‐uncertainty; SOD, self‐other differentiation.

*t* Tests for gender differences showed significant differences concerning all the variables of interest, except for CEA. Specifically, males scored higher than females on self‐other differentiation with a medium effect size. In contrast, females scored higher than males on problematic SNS use and on reflective functioning uncertainty with a small effect size.

Pearson's *r* correlations between age and all the psychological measures are reported in Table 2. Associations between sex and the other variables were assessed via point‐biserial correlations. Problematic SNS use was significantly and positively associated with CEA and reflective functioning uncertainty and significantly and negatively associated with adequate self‐other differentiation.

**Table 2:** Correlations between problematic social networking sites use, childhood emotional abuse, self‐other differentiation, and reflective functioning uncertainty

	1.	2.	3.	4.	5.	6.
1. Age	–					
2. Sex	0.003	–				
3. CEA	0.039	0.040	–			
4. SOD	0.004	−0.254***	−0.159***	–		
5. RFu	−0.027	0.111***	0.193***	−0.398***	–	
6. IAT‐SN	−0.037	−0.125***	0.229***	−0.276***	0.327***	–

Abbreviations: CEA, childhood emotional abuse; IAT‐SN, Internet addiction test social network; RFu, reflective functioning‐uncertainty; SOD, self‐other differentiation.

*** *p* < 0.001.

**Figure 2:**
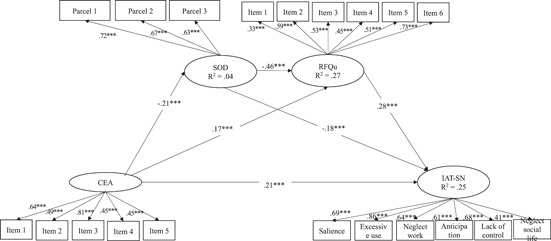
Standardized estimates for the mediation model. CEA, childhood emotional abuse; IAT‐SN, Internet addiction test social network; RFu, reflective functioning‐uncertainty; SOD, self‐other differentiation

SEM was conducted to test the mediating effect of self‐other differentiation and reflective functioning uncertainty on the relationship between CEA and problematic SNS use. Due to skewness and kurtosis values of EA over the threshold of 2, which indicate non‐normality of data distribution, the robust maximum likelihood estimator (MLR) was used for the measurement and full models. Furthermore, given that gender differences emerged in the *t *tests, we tested two separate models for males and females. Both models showed an adequate goodness of fit with observed data, MLR *χ* ^2^_(162)_ = 293.21, *p* < 0.001; RMSEA = 0.036; CFI = 0.95; SRMR = 0.04 for males; MLR *χ* ^2^_(162)_ = 412.03, *p* < 0.001; RMSEA = 0.048; CFI = 0.92; SRMR = 0.05 for females. Therefore, we decided to report the findings of the total sample. The analysis of the modification indices identified that errors of item 4 and item 5 of the CTQ and the Salience and Anticipation subscales of the IAT‐SN were correlated. Since these variables examined highly related dimensions of the investigated constructs, their errors were allowed to covariate in the model (*r* = 0.38, *p* < 0.001 and *r* = 0.92, *p* < 0.001, for CTQ items and IAT‐SN subscales, respectively). The full mediation model, including both the measurement and structural components, reported adequate indices of fit: MLR *χ* ^2^_(162)_ = 526.50, *p* < 0.001, RMSEA = 0.041, CFI = 0.94, SRMR = 0.04. The results of the structural part of the model are depicted in Figure 2.

With regard to direct effects, the results indicated that higher levels of CEA were directly associated with lower levels of SOD and higher levels of reflective functioning uncertainty. Higher levels of SOD were, in turn, associated with lower levels of reflective functioning uncertainty. Finally, the antecedent and mediator variables were all directly associated with the problematic SNS use scores: Higher levels of CEA, lower levels of SOD, and higher levels of reflective functioning uncertainty were all associated with greater problematic SNS use.

As for the indirect effects, the mediation path from CEA to problematic SNS use through inadequate SOD was significant, as well as the path including only reflective functioning uncertainty as a mediator. The full sequential mediation path, including both inadequate SOD and reflective functioning uncertainty as mediators, showed a small but significant indirect effect.

## 4 DISCUSSION

The present study found a significant and positive association between CEA and problematic SNS use in adolescents. The study also showed that the relationship between CEA and problematic SNS use was partially mediated by deficient self‐other differentiation and reflective functioning. However, it must be noted that here, we use the term “mediation” in its statistical sense, given that the cross‐sectional design of the study excludes the possibility of testing for the causal direction of associations.

The finding of a positive and strong association between problematic SNS use and CEA is consistent with the results reported by Kircaburun et al. (2019a) and provides support for the compensatory model of PIU (Kardefelt‐Winther, 2014a). Accordingly, some adolescents with a history of CEA may develop problematic SNS use as a way of coping with the consequences of CEA, such as a poor sense of self‐worth and a tendency to be overwhelmed by negative affect and aversive emotions. This finding also confirms that traumatic experiences in childhood other than interpersonal physical violence (e.g., child physical or sexual abuse) need to be considered in relation to Internet‐related disorders (Dalbudak et al., 2014; Durkee et al., 2012).

The finding of a positive association between problematic SNS use and deficient self‐other differentiation suggests that adolescents with an unstable sense of self and problems in interpersonal relationships may overuse online interactions (Kraut et al., 1998) to mitigate these difficulties (Assunção et al., 2017). For example, adolescents with low levels of self‐other differentiation may use instant messaging (e.g., Facebook Messenger) to get immediate feedback on relevant features of their personality (Shapiro & Margolin, 2014). The finding of a positive association between problematic SNS use and deficiencies in reflective functioning suggests that adolescents with a poor understanding of their own and others' mental states are at higher risk of problematic SNS use. In fact, SNS combine a sense of connectedness with physical distance (Allen et al., 2014), which could be appealing for individuals with a history of CEA because it might provide feelings of safety via the control of intimacy in interactions and relationships that SNS allow for.

The results of the structural equation modeling show that CEA is positively associated with a lower self‐other differentiation, which is, in turn, related to lower reflective functioning and higher problematic SNS use in adolescents. In other words, some adolescents who overuse online relationships have a history of CEA, unclear self‐other boundaries, and inadequate reflective functioning ability. A poorly differentiated sense of self may lead to reflective functioning impairments in some adolescents and may even result in what was termed “egocentric communication” (Wichstrøm & Holte, 1991). That is, if an individual expects to understand others as a mere reflection of his or her expectations, he or she will not find it necessary to make an effort to understand the other person's perspective. Moreover, because of their unstable self‐other boundaries and difficulties in understanding others’ mental states, such an individual may be more likely to have unstable interpersonal relationships offline and perceive an online environment as a safe place to connect with other people. Thus, SNS use may help an individual suffering from these deficiencies in reflective functioning to avoid excessive and potentially threatening closeness with others (Liu et al., 2013), making it easier to initiate relationships (e.g., by receiving immediate attention and validation from others via “likes”; Dumas et al., 2017). However, inordinate reliance on online relationships may contribute to the development of problematic SNS use (Marino, Gini, et al., 2018; Saiphoo et al., 2020).

From a theoretical standpoint, our results are consistent with an attachment‐oriented psychodynamic framework (Schimmenti & Caretti, 2010). Within this framework, the negative internal working models of the self, the other, and self‐other relationships that occur as a consequence of CEA affect one's subsequent interpersonal relationships, including various offline and online interactions. Furthermore, the internalization of early negative relationships with caregivers in the context of CEA is associated with difficulties in disclosing painful experiences and managing psychic pain (Schimmenti, 2012). More specifically, according to Schimmenti and colleagues (Schimmenti & Caretti, 2010, 2017; Schimmenti et al., 2012), online addictive behaviors may have a defensive function, as they may protect the individual against the memories of CEA, that is, against the “psychic pit in which feelings, states of mind and piece of one's own self are buried and lost in oblivion” (Schimmenti & Caretti, 2010, p. 127).

Adolescents with a history of CEA may also use SNS excessively to escape from an unpleasant reality and thereby manage their dysregulated sense of self and painful mental states. As demonstrated by previous studies (e.g., Di Blasi et al., 2019; Musetti et al., 2019), these online escapist behaviors often create more interpersonal disconnection and isolation, with individuals ultimately failing to respond effectively to their own emotional needs, which can reactivate traumatic memories (Schimmenti & Caretti, 2016). Accordingly, research shows that some anxiously attached individuals make a lot of effort to maintain relationships via SNS (D'Arienzo et al., 2019; Lin, 2016) as compensation for the lack of affection they receive from family members (Rao & Madan, 2012).

In agreement with previous findings (e.g., Hou et al., 2017; Kuss & Griffiths, 2017), we found that females reported higher levels of problematic SNS than males. One explanation for this finding is that males may be more likely to overuse the Internet for other purposes, like gaming (e.g., Dindar, 2018; Kaur & Kaur, 2017), whereas females tend to use the Internet for relational purposes and may be more likely to develop problematic SNS use to compensate for unmet social needs offline (Barker, 2009) and regulate their mood (Boursier et al., 2020). Moreover, our findings are in agreement with studies that reported a stronger association between engagement in social media use and depressive mood, low self‐esteem, and other psychological symptoms among females than males (McCrae et al., 2017; Nowland et al., 2018; Raudsepp & Kais, 2019).

Our findings have important clinical implications. Mentalization‐informed interventions (see Malda‐Castillo et al. (2019) for a review) could help adolescents with problematic SNS use become more aware of self‐other boundaries and their mental states, improve their ability to regulate emotions, and discourage maladaptive strategies for managing internal states, such as substance use and addictive behaviors. This could be achieved through a better understanding of the developmental roots of a dysregulated sense of self and painful mental states within a safe therapeutic relationship. Ultimately, psychological intervention should focus on the appropriate processing of traumatic experiences (Wéry et al., 2018) that would allow for the development of a more coherent and stable sense of self.

The findings of this study should be interpreted in light of several limitations. Data were collected from a limited number of students whose high schools gave them permission to participate in the study, thus limiting the generalizability of the results. Moreover, the cross‐sectional nature of the data precludes consideration of the direction of the effect; therefore, no definitive conclusions can be drawn about the stability of the mediated effects found in this study. The mediation model was based on theory, which does not necessarily exclude reverse causality, although CEA can theoretically be assumed to be the antecedent variable. Longitudinal studies are therefore needed to clarify the relationships among the variables and further examine the proposed multiple mediation model. Due to the retrospective nature of this study, our findings may be subject to self‐serving or recall bias. Likewise, our findings are based entirely on self‐report measures, which may have led to biased reporting. Also, we did not assess the type of SNS used by the participants, and problematic SNS use was measured by an adaptation of the IAT, which did not allow us to distinguish between problematic SNS use and high involvement (i.e., intensive but healthy use) in social networks. Finally, our findings may have been affected by other variables not examined here, such as other forms of maltreatment that often co‐occur with CEA in abusive families (Petrone et al., 2012) or factors like insecure attachment, loneliness, and alexithymia. This calls for further studies in clinical and nonclinical samples of adolescents, especially given the importance of problematic SNS use in adolescence.

These limitations notwithstanding, we conclude that ours is the first study to demonstrate the relationships between CEA, low self‐other differentiation, uncertain reflective functioning, and problematic SNS use in adolescence. The study contributes to the body of knowledge by suggesting that adolescents with a history of CEA also seem to have difficulties with experiencing a separate sense of self in interpersonal relationships and, in turn, problems with understanding their own mental states and the mental states of others. These factors may contribute to problematic SNS use, which can then develop as an attempt to regulate the sense of self and painful emotional states linked to traumatic memories. Such dynamics call for tailored clinical interventions.

## CONFLICT OF INTERESTS

The authors declare that there are no conflict of interests.

## ETHICS STATEMENT

School approval and parental written informed consent were obtained prior to before students’ participation in the study.

### PEER REVIEW

The peer review history for this article is available at https://publons.com/publon/10.1002/jclp.23138

## Data Availability

The data that support the findings of this study are available from the corresponding author upon reasonable request.
